# Parametric modeling and engineering application of the high-speed continuous beam bridge based on Building Information Modeling

**DOI:** 10.1371/journal.pone.0310310

**Published:** 2024-09-10

**Authors:** Hanwen Jiang, Zhiliang Ma, Yi Li, Daixuan Wu, Han Sai, Kun Zhang, Kun Chen

**Affiliations:** 1 China Construction Communications Engineering Group Co., Ltd., Beijing, China; 2 School of Civil Engineering, Tsinghua University, Beijing, China; University of Vigo, SPAIN

## Abstract

To improve the informatization and intelligence level of high-speed railway (HSR) bridge construction, a parametric modeling method for continuous beam bridges based on Building Information Modeling (BIM) is proposed in this study. By this method, the parametric families of continuous beam components and key construction machinery are established, and the rapid modeling of overall continuous beam bridge and the simulation of critical construction process are realized as well. Taking the Caoxian-Shangqiu bridge of Xiong’an-Shangqiu HSR as a case study, the parametric modeling method is applied to conduct the engineering application on the prestressed duct layout and rebar clash detection. The results indicate that the modeling efficiencies of HSR continuous beam bridge and construction machinery are significantly increased by the established parametric modeling method. Based on the BIM model of continuous beam bridge, the improvement in the precision of prestressed duct layout and the elimination of rebar clash points can be achieved. The research achievement can guide the visualization of construction disclosure, enhance construction efficiency, and provide reference and technical support for the construction management and control of HSR continuous beam bridges.

## 1. Introduction

### 1.1 Literature review

With the rapid development of the High-speed Railway (HSR) in China, the development of transportation and civil economy have been boosted. The application of bridges in HSR is to ensure the smoothness and safety of operation. Taking the Beijing-Shanghai HSR and Shanghai-Hangzhou HSR as examples, the mileage of bridges accounts for over 80% and 90% of the total mileage, respectively. Long-span prestressed concrete bridge is an important structural form of HSR bridges, which has the advantages of excellent mechanical properties, high smoothness, and stability. However, the traditional construction management mode of the HSR continuous beam bridge restricts the development of bridge construction towards informatization and digitalization construction. The level of bridge construction management needs to be improved.

In recent years, the development of Building Information Modeling (BIM) has provided an efficient method for promoting the informatization level of bridge construction management [1–4]. Many experts and scholars have conducted related research. Eastman et al. [5] thoroughly discuss how BIM facilitates detailed modeling, which significantly improves the visualization and pre-construction analysis of bridge projects. This precision in planning is instrumental in preempting construction issues, thus optimizing resource utilization and cost. Girardet and Boton [6] develop a parametric file which is able to establish all types of bridges from a single parametric file. The proposed file applies a parametric algorithm for bridge modeling elements in design software, and also for the generation and analysis of bridge model in structural analysis software. Scianna et al. [7] present a case study illustrating the integration of an Internet of Things (IoT) system with a BIM framework. To monitor the deflection at the centerline of a bridge beam, a scaled-down bridge model, which can manage multiple bridge construction projects intelligently with promoted efficiency, is recreated and analyzed within a BIM environment. Wang [8] explores the integration of BIM technology to develop an intelligent platform for managing bridge construction. It delves into the implementation of vibration signal processing, feature extraction, fault diagnosis, and wireless communication functionalities within this platform. In the context of bridge engineering, BIM and Geographic Information System (GIS) applications have become increasingly popular. They aid in heterogeneous aspects like simulation, visualization, and decision-making processes, including the selection of bridge locations and the maintenance of existing structures [9]. The current bridge inspection practices are usually based on manual paper-based data collection methods, which greatly hinder the transfer of knowledge gained throughout the lifecycle of the asset, ultimately impeding the assessment of inspectors or engineers. Chan et al. [10] seek to address the shortcomings of existing methods by introducing a conceptual framework that enhances the reliability and efficiency of present bridge asset management techniques. This is achieved through the incorporation of BIM, along with advanced computing and imaging technologies. As a pivotal tool in bridge inspections, BIM demonstrates substantial capabilities, especially when merged with laser scanning and keypoint-based texture recognition methods. This combination enables the identification of various defects in bridge structures, including cracking, corrosion, and settlement. Wang et al. [11] develop a method for automatically measuring the dimensions of precast concrete bridge deck panels and generating as-built BIM models. The process starts with scan planning to identify the best scanner positions for data collection. Next, the scan data of the panel are gathered and refined by eliminating noise and aligning multiple scans in a unified coordinate framework. Using this processed data, the as-built geometries of panel are determined. Finally, an as-built BIM model is constructed using these measured geometries. The effectiveness is verified using a lab-scale sample and a full-scale precast concrete bridge deck panel. The results demonstrate that this approach can measure full-scale panels with a 3 mm accuracy precisely and efficiently and create as-built BIM models autonomously. Hüthwohl et al. [12] conduct an exploratory study resulting in an information model using the Industry Foundation Classes (IFC) framework, aiming at organizing and standardizing the storage of inspection data for reinforced concrete (RC) bridges efficiently. Their model facilitates data sharing and comparison. The team demonstrates the IFC’s latest version’s capability to integrate defect information and images in three steps: identifying defect types and properties from bridge inspection manuals, modeling these defects and their relationships, and integrating these models into the IFC framework. A prototype is developed to test automated data sharing and comparison, enhancing the knowledge base for bridge performance monitoring. Lee et al. [13] develop an automated system for extracting bridge design parameters, aiming to streamline the scan-to-BIM process. This system operates in three stages: noise reduction, 3D transformation, and parameter extraction. It is tested on the Osong test track’s fifth bridge. The system developed in this study successfully extracts the design parameters of the bridge from the point cloud data (PCD) automatically, resulting in a 0.8% error rate. Kaewunruen et al. [14] propose a 6D modeling approach for bridge projects, combining 3D models with time schedules, cost estimates, and carbon footprint analysis throughout the project’s lifecycle. The study finds that raw materials are the main source of embodied carbon emissions. By developing the 6D BIM model early in the project lifecycle, it allows stakeholders to work together within the BIM environment to achieve more sustainable and cost-effective results from the outset. Byun et al. [15] establish a BIM-based Bridge Management System (BMS) to enhance the sustainability of bridge maintenance. The system consists of creating a comprehensive maintenance data schema and information system, compiling details on safety diagnosis, repair, strengthening, lifespan, and valuation. A prototype of this innovative BMS is implemented for a real bridge in Korea, which aims to improve current maintenance management practices, reduce costs, and minimize information loss. Deng et al. [16] introduce a method for managing and visualizing bridge health monitoring information using BIM. It integrates visual warning and information management plugins into Revit software through its Application Programming Interface (API). The method includes creating a virtual sensor system and a monitoring information model for front-end visualization. A database-driven platform is established to link with the BIM model for storage, viewing, and analysis directly from Revit. The approach enhances the integration and visualization of monitoring data. The implementation is exemplified through the health monitoring project of Ge Xian Bridge in China. Zhou [17] presents a novel bridge management and maintenance approach using a BIM system. It aims to develop an intelligent model for real-time monitoring and management of bridge issues, enhancing safety and reducing accidents. The system can enable data-driven, scientific methods for bridge restoration, ensuring public safety. By combining BIM technology, the approach creates a precise and visible model, improving the efficiency of bridge management and maintenance significantly [18, 19].

Based on the literature review, the typical applications of BIM technology in bridge engineering primarily include BIM modeling and forward design of bridges, interaction with finite element software, construction process simulation, visualized disclosure, and digital monitoring and maintenance, etc [16, 20, 21]. Key features of BIM modeling and forward design of bridges are parametric design, component associativity, and parameter-driven shape design. However, the BIM model of bridges has the characteristics of few varieties, large quantities, and complicated locations. The one-by-one modeling method has the problems of low utilization, large modeling workload, and low information transfer efficiency [22, 23], and the advantages of BIM parameterization and information integration cannot be fully utilized as well.

Therefore, the parametric modeling application in the bridge construction process needs to be further studied. Parametric modeling is defined as a process that uses algorithmic thinking to encode design intent and relationships through parameters and constraints. This method allows for flexibility and adaptability in design modifications. The main types of parametric modeling utilized in this study are geometric modeling, which focuses on the precise control of shapes and forms of bridge components, and functional modeling, which integrates performance criteria and functional requirements to optimize the design process. The parametric modeling allows for rapid adjustments and fine-tuning of the bridge components by altering the defined parameters. This results in significant improvements in design accuracy and efficiency. By employing parametric modeling, any design modifications are automatically updated throughout the model, thus reducing errors and enhancing coordination among different design elements. The characteristics of BIM involved in parametric modeling include data integration, collaborative capabilities, and enhanced visualization. The BIM technology also enables the integration of various data sources into a unified model, allowing for comprehensive analysis and management of project information. The collaborative nature of BIM supports seamless communication and coordination among different stakeholders, ensuring that parametric changes are consistently reflected across all aspects of the project. Furthermore, the advanced visualization tools of BIM help in identifying potential design issues early in the process, thereby improving overall project outcomes.

### 1.2 Aims and objectives

To develop a rapid parametric modeling method for HSR continuous beam bridges using BIM, aimed at improving the informatization and intelligence level of bridge construction. The objectives of this study are presented as follows:

- To establish parametric families for HSR continuous beam components and key construction machinery, thereby enhancing the efficiency and precision of the modeling process.
- To apply the developed parametric modeling method in the construction of the Caoxian-Shangqiu section of the Xiong’an-Shangqiu HSR, focusing on critical construction aspects such as prestressed duct layout and rebar clash detection.
- To evaluate the impact of the parametric modeling approach on construction efficiency and the management of construction data, contributing to the advancement of digital construction methods in the HSR bridge sector.

Therefore, a parametric modeling method of HSR continuous beam bridge is developed based on BIM in this study, and the Caoxian to Shangqiu bridge of Xiong’an to Shangqiu HSR is taken as engineering background. And the engineering application study of the construction machinery, prestressed duct layout, rebar clash detection, the overall model of construction site, and so on, are carried out. This work can provide the construction reference and basis for improving the informationalized level and construction efficiency of HSR continuous beam bridges.

## 2. The parametric modeling of HSR continuous beam bridge

### 2.1 Theoretical foundations

Parametric modeling and BIM are transforming bridge design and construction, offering unprecedented precision and efficiency. This section delves into the core theoretical concepts and their practical implications in bridge engineering.

Parametric modeling involves the representation of data and dimensions through parameters, which can be adjusted to explore various design alternatives quickly. This approach is particularly beneficial in bridge construction, where design flexibility and efficiency are paramount. Recent advancements in this field have led to the development of sophisticated models that can dynamically adjust to changes, thus improving design iterations and decision-making processes [24].

BIM extends beyond traditional 3D modeling by incorporating 4D (time) and 5D (cost) elements [25–27], which are crucial for the lifecycle management of bridge projects. BIM’s integration into bridge construction facilitates enhanced collaboration among all stakeholders and improves the management of the project’s structural data, from initial design through to construction and maintenance [28].

The synergy between parametric modeling and BIM technologies enables more resilient and adaptable bridge designs. BIM models can be applied in the construction period to guide the visualization of construction disclosure, enhance construction efficiency, and provide reference and technical support [29].

Based on the literature reviews, it can be found that the parametric modeling of BIM has immense potential for application in bridge construction. However, recent researches are lacking, so it is critical to carry out the research of the parametric modeling method of HSR continuous beam bridge to improving the efficiency and quality of construction.

### 2.2 Methodology

Aiming at the disadvantages of poor reuse performance and low modeling efficiency of the one-by-one modeling method of HSR continuous beam bridge, a rapid parametric modeling method based on BIM is proposed in this study. The method is characterized by the parameterization of the profile and material information of generic bridge components. By altering key parameters, rapid modeling of similar components is achieved, thereby enhancing the overall modeling efficiency of HSR continuous beam bridges.

The parametric modeling carried out is based on the China-made BIM platform, which supports extensive customization through its parametric design capabilities. And the parametric modeling is conducted by attaching the self-programming plugin to the BIM software [30–33].

The development of our parametric modeling method involved creating customized families through the plugin in the BIM platform, each tailored to represent different structural components of HSR continuous beam bridges, thereby enabling rapid adjustments and iterations during the modeling phase. The Caoxian-Shangqiu bridge is taken as the modeling instance, and the BIM model is established by the parametric modeling method. Based on the model, the prestressed duct layout and rebar clash detection are conducted to improve efficiency and quality during the construction period.

**Fig 1:**
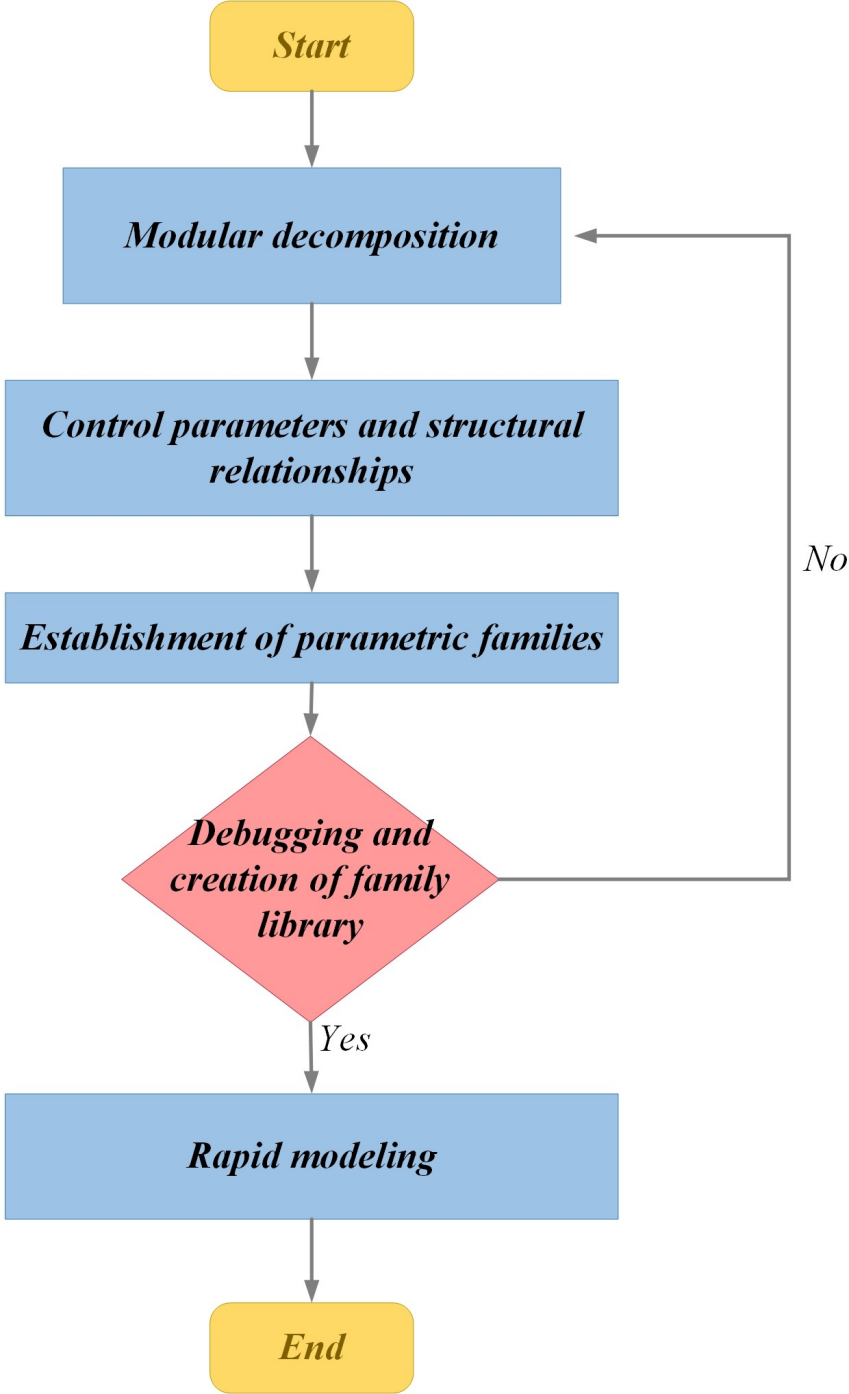
The process of parametric modeling of HSR continuous beam bridge.

The process of this method is illustrated in Fig 1 and primarily encompasses the following steps:

1. Modular decomposition
  - The bridge structures and construction machinery are modularly decomposed according to different principles such as construction methods and stages.
  - This modular decomposition enables focused parameterization and efficient modeling.
2. Control parameters and structural relationships
  - Control parameters such as dimensions, profiles, materials, and quantities are identified and clearly defined for each component.
  - The structural relationships between components are clarified to ensure accurate and efficient modeling.
3. Establishment of parametric families
  - Preliminary parametric families for each bridge component and construction machinery are created.
  - These families include detailed parameters for geometric dimensions, material properties, and spatial relationships.
4. Debugging and creation of family library
  - Each parametric family undergoes rigorous debugging to ensure accuracy and reliability.
  - A comprehensive parametric family library is established, covering all necessary components for the HSR continuous beam bridge.
5. Rapid modeling

- The overall model of the HSR continuous beam bridge is rapidly constructed using the parametric family library.
- Control parameters allow for quick adjustments and iterations, significantly enhancing modeling efficiency.

**Fig 2:**
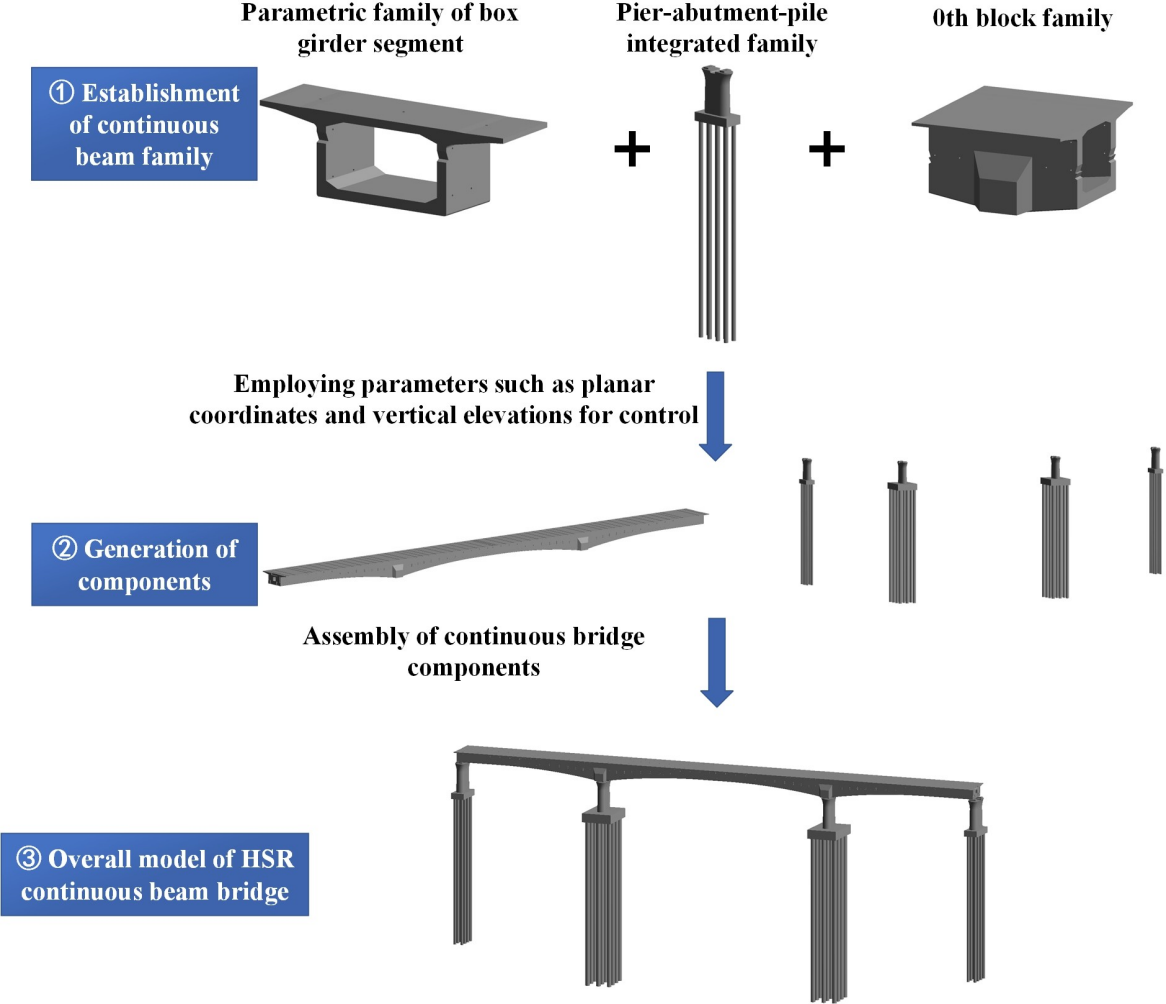
The rapid modeling process of continuous beam bridge.

Based on the modeling process mentioned above, the components and assemble process of HSR continuous beam bridge model are detailed in Fig 2.

### 2.3 Engineering background

**Fig 3:**
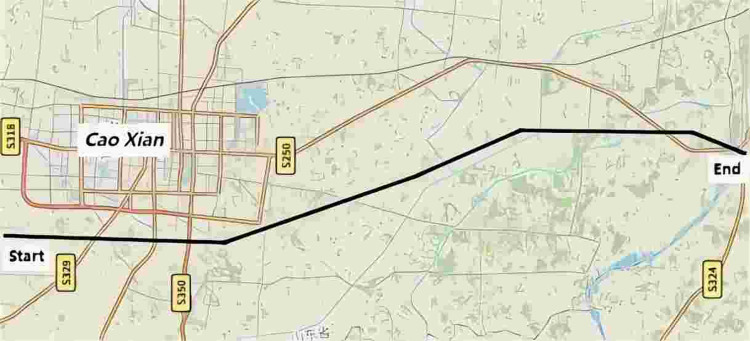
The 14^th^ section of Xiong’an-Shangqiu HSR (note that the figure uses map data from OpenStreetMap and their data sources; see openstreetmap.org/copyright).

**Fig 4:**
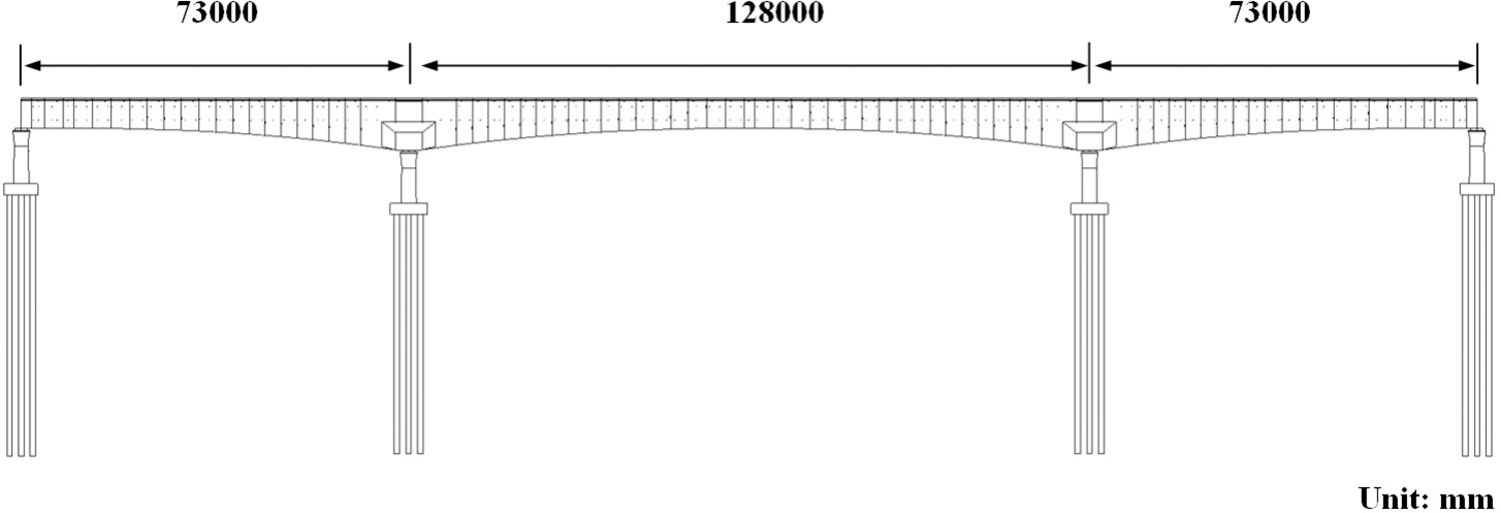
The schematic illustration of bridge construction.

The Caoxian-Shangqiu Grand Bridge in the 14^th^ section of Xiong’an-Shangqiu HSR is selected as the modeling instance. The length of this section is 38.452km, and the planning graph is illustrated in Fig 3. The span arrangement of the Caoxian-Shangqiu Grand Bridge is designed as a continuous beam measuring (73+128+73) m. The superstructure of the bridge is constituted by prestressed concrete continuous box girder. It is distinguished by its straight webs, a variable cross-section, and a variable height. And the schematic illustration of bridge construction can be seen in Fig 4.

It can be observed from Fig 4 that the section height and bottom plate thickness of each segment of the continuous beam vary in accordance with the curvature of the bridge bottom. Therefore, it is necessary to enhance the efficiency of the modeling process by segmented modeling. The geometric relationships of the segment dimensions can be adjusted through the setting of control parameters, thereby facilitating rapid modeling of the bridge components and enhancing work efficiency.

### 2.4 The parametric family of bridge structure

#### 2.4.1 The parametric family of box girder segment

**Fig 5:**
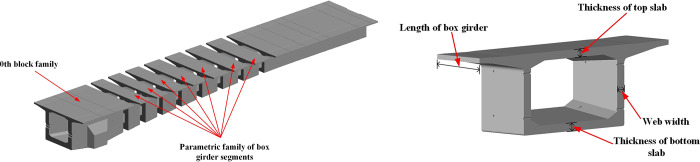
The parametric family of (a) the box girder segments and (b) the structure model.

The single-box and single-chamber box girders of the continuous beam bridge are divided into multiple segments. Apart from the 0th block, each segment has an identical structural form with consistent thickness in the top slab and web slab, differing only in size. Therefore, a parametric family model of the box girder segments can be established, excluding the 0th block, which still needs to be modeled separately. To facilitate the parametric modeling of box girder segments, certain simplifications are supposed to be put forward. For instance, the bidirectional cross-slope on the top of the box girders and the internal teeth blocks are not considered. The section height and bottom thickness of each segment are treated as the linear variation. Based on that, the box girder length, section height, top slab thickness, bottom slab thickness, and web width, along with other geometric information, are utilized as modeling parameters to establish a parametric family of box girder segments, as illustrated in Fig 5.

The specific modeling steps can be summarized as follows:

1. Set up layout reference planes in the ’Family’ editor;
2. Add model dimension annotations and labels according to the characteristics of the drawings;
3. Draw geometric shapes and lock them to the reference planes;
4. Configure the ’Family’ properties, including parameter naming, parameter calculation formulas, parameter units.

#### 2.4.2 The parametric family of the substructure

**Fig 6:**
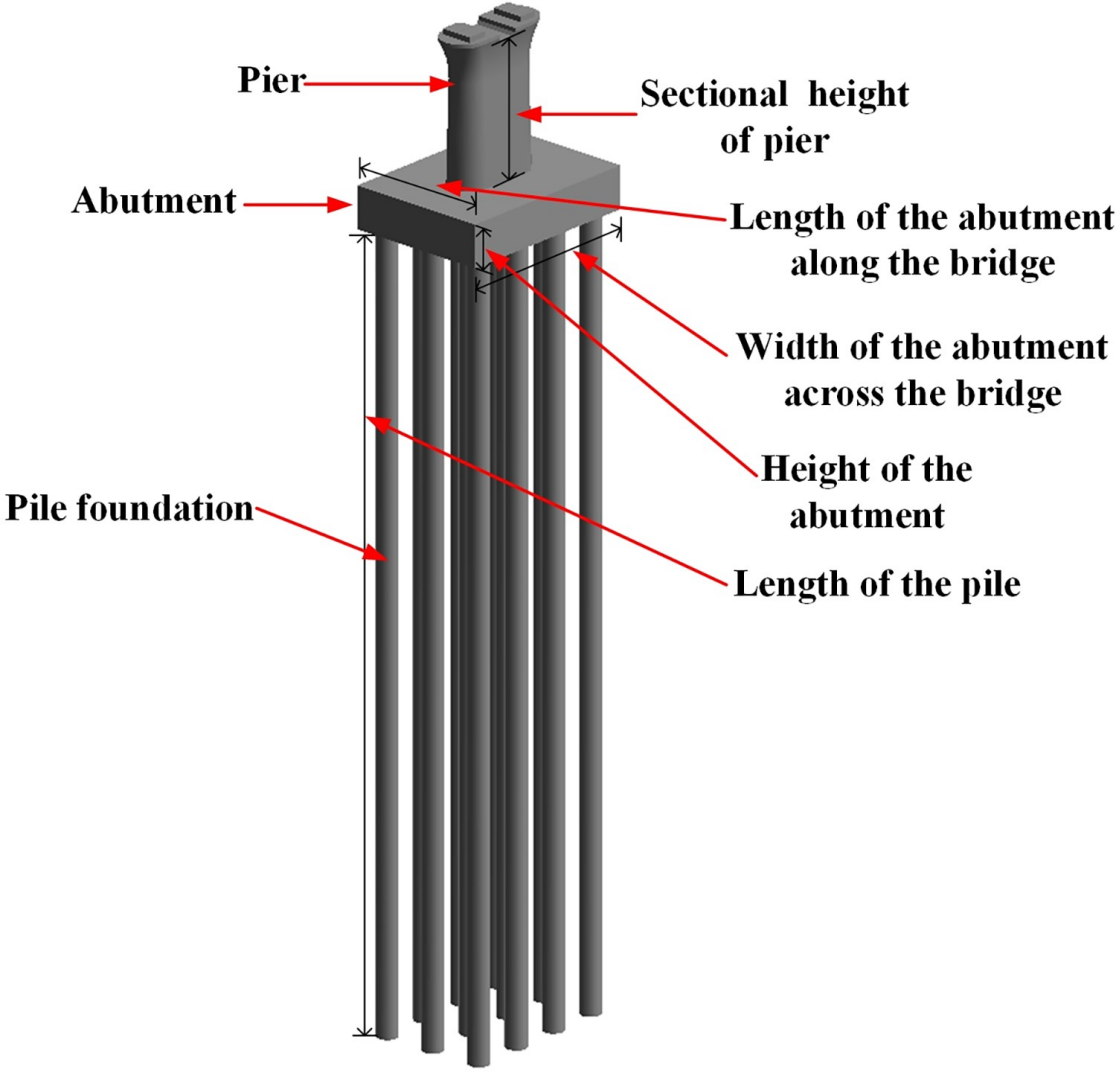
The pier-abutment-pile integrated family.

The substructure of continuous beam bridges is mainly composed of piers, abutments, and pile foundations. And a uniform structural design or a symmetrical structural design is usually employed for bridge piers. Based on the characteristics of the bridge design drawings, an integrated family of piers, abutments, and pile foundations can be chosen for modeling to economize on the modeling time of the substructure. This integrated family consists of round-ended solid piers, rectangular abutments, and bored piles. The modeling parameters considered in this section are the sectional height of the round-ended solid piers, the length of the abutment along the bridge, the width of the abutment across the bridge, the height of the abutment, the length of the piles, the diameter of the pile foundation, and the number of piles. The integrated family is illustrated in Fig 6.

The specific modeling steps can be summarized as follows:

1. Use uniform structural design or symmetrical design principles for modeling.
2. Set sectional height, abutment length and width, pile length, diameter, and quantity as changeable parameters.

#### 2.4.3 The parametric family of prestressed steel strands

In the entire process of bridge modeling, the modeling of prestressed steel strands is identified as the task with the greatest workload. It is observed that not only is the number of these strands considerable, but the sizes and positions are also subject to continuous variation in accordance with the changing cross-sections of the box girder segments. By employing the parametric modeling approach, where the cross-section of the box girder segments serves as the reference plane, a substantial reduction in both modeling workload and time can be realized.

**Fig 7:**
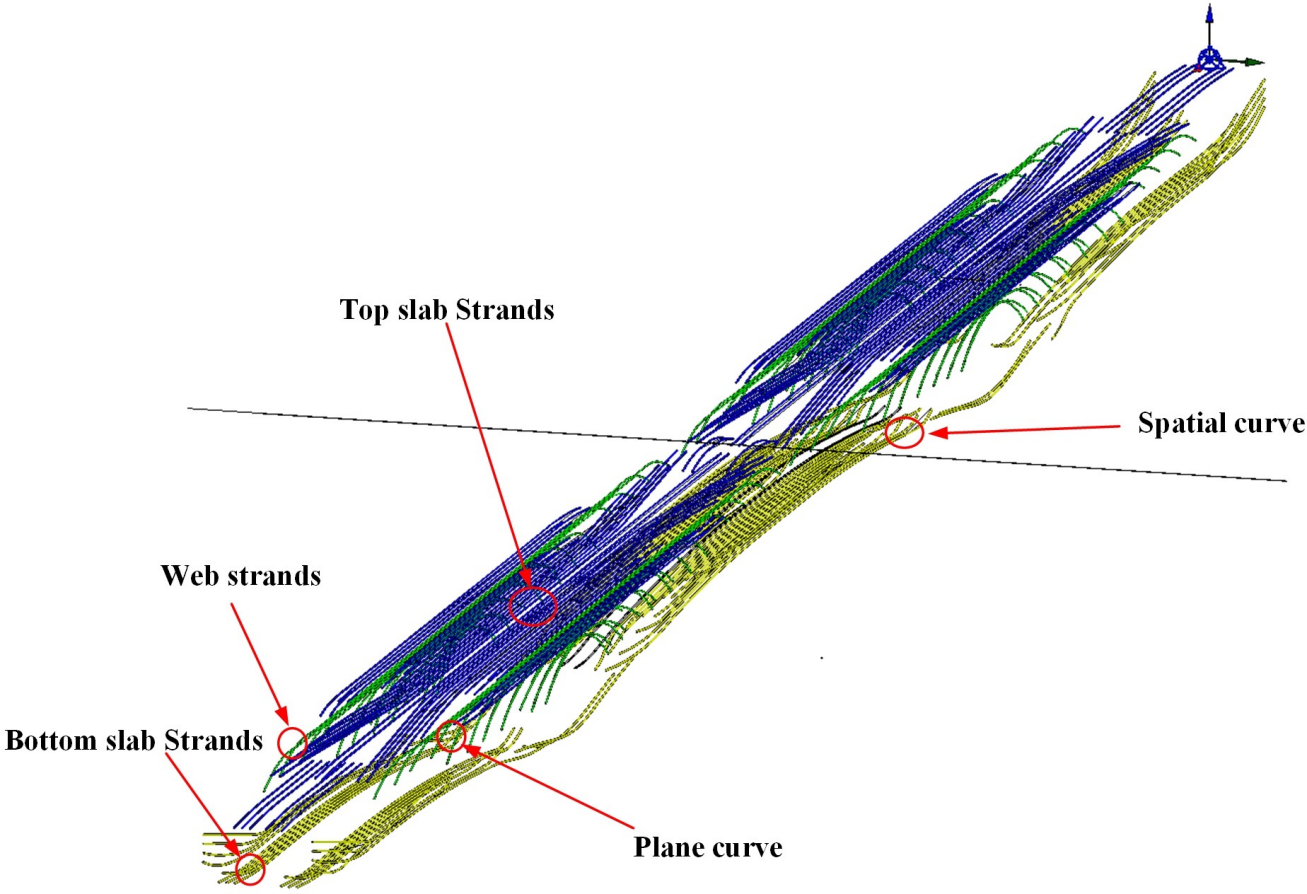
The parametric family of prestressed steel strands.

Fig 7 illustrates the established parametric family of prestressed steel strands. Depending on the layout form, there are two modeling methods: 1) For prestressed steel strands laid out in a planar curve, the strand line shape can be directly imported, and modeling is conducted using conventional families; 2) For strands laid out in a spatial curve, a spatial curve is obtained by performing difference operations on the planes extended from the planar and vertical curves. After establishing the line shape of steel strands, modeling is then carried out.

### 2.5 The parametric family of the construction machinery

#### 2.5.1 The parametric family of the 0th block support

The construction of the 0th block support, as a focal project in the construction of continuous beam cantilever, is critical to the entire continuous beam construction. By analyzing the structural parameters and the layout characteristics of the support structure, the information for model creation can be categorized into two types structural information and layout information. Structural information primarily represents the material types and structural parameters used for the support components, while layout information denotes the spatial position information of each component. Using the top center point of the bearing platform as the reference point, the components such as square timber, adjustment pad beams, side mold longitudinal beams, large cross beams, and steel tube columns are sequentially arranged from top to bottom according to the layout elevation.

**Fig 8:**
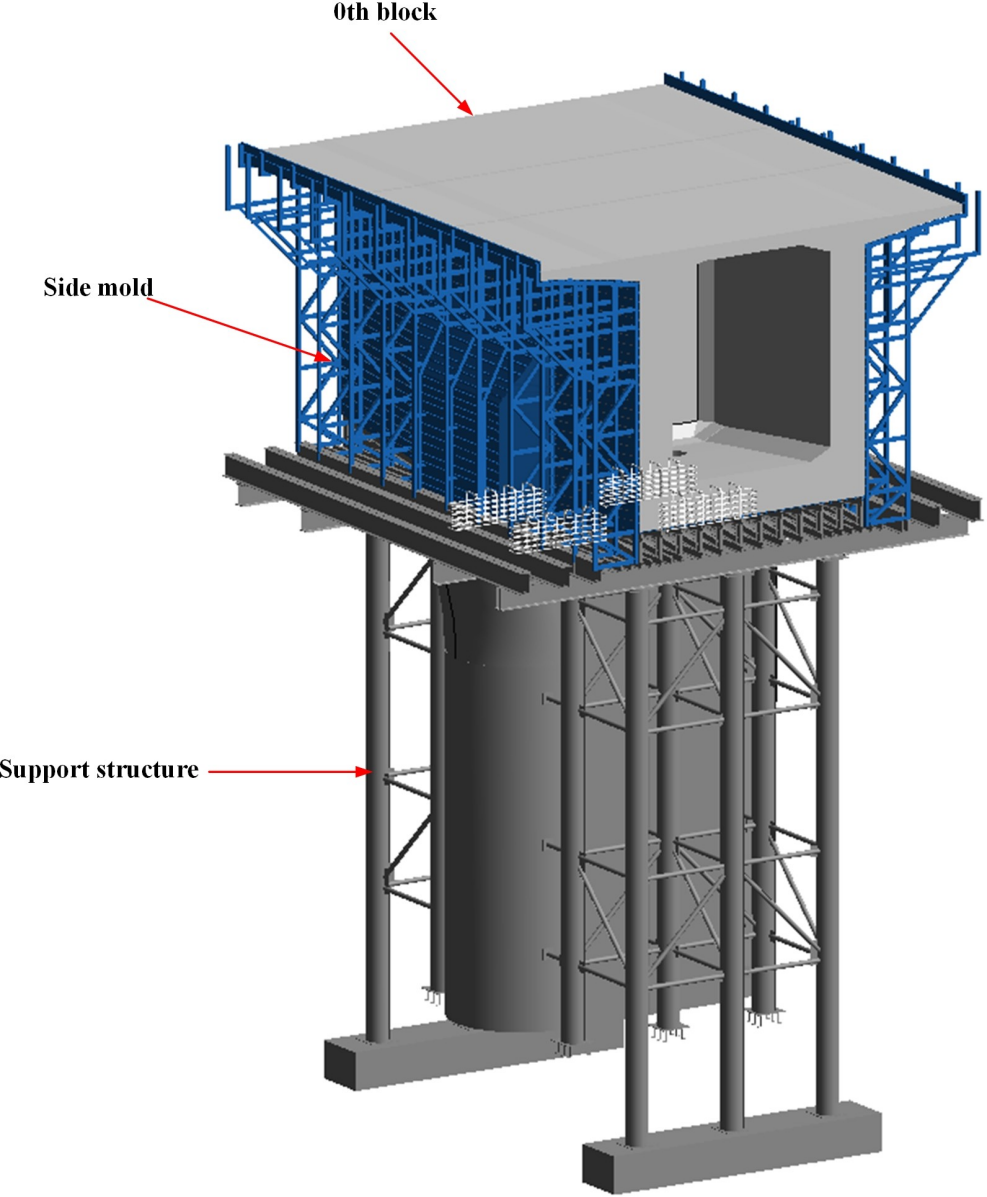
The integrated model of the main structure, support structure, and side mold.

Utilizing the aforementioned method, the parametric modeling of the 0th block, bridge piers, bearing platform, support structure, and side mold model is conducted. The components are then assembled based on position information, resulting in an integrated BIM model of the main structure, support structure, and side mold. The integrated mode is illustrated in Fig 8.

#### 2.5.2 The parametric family of the Bailey truss

**Fig 9:**
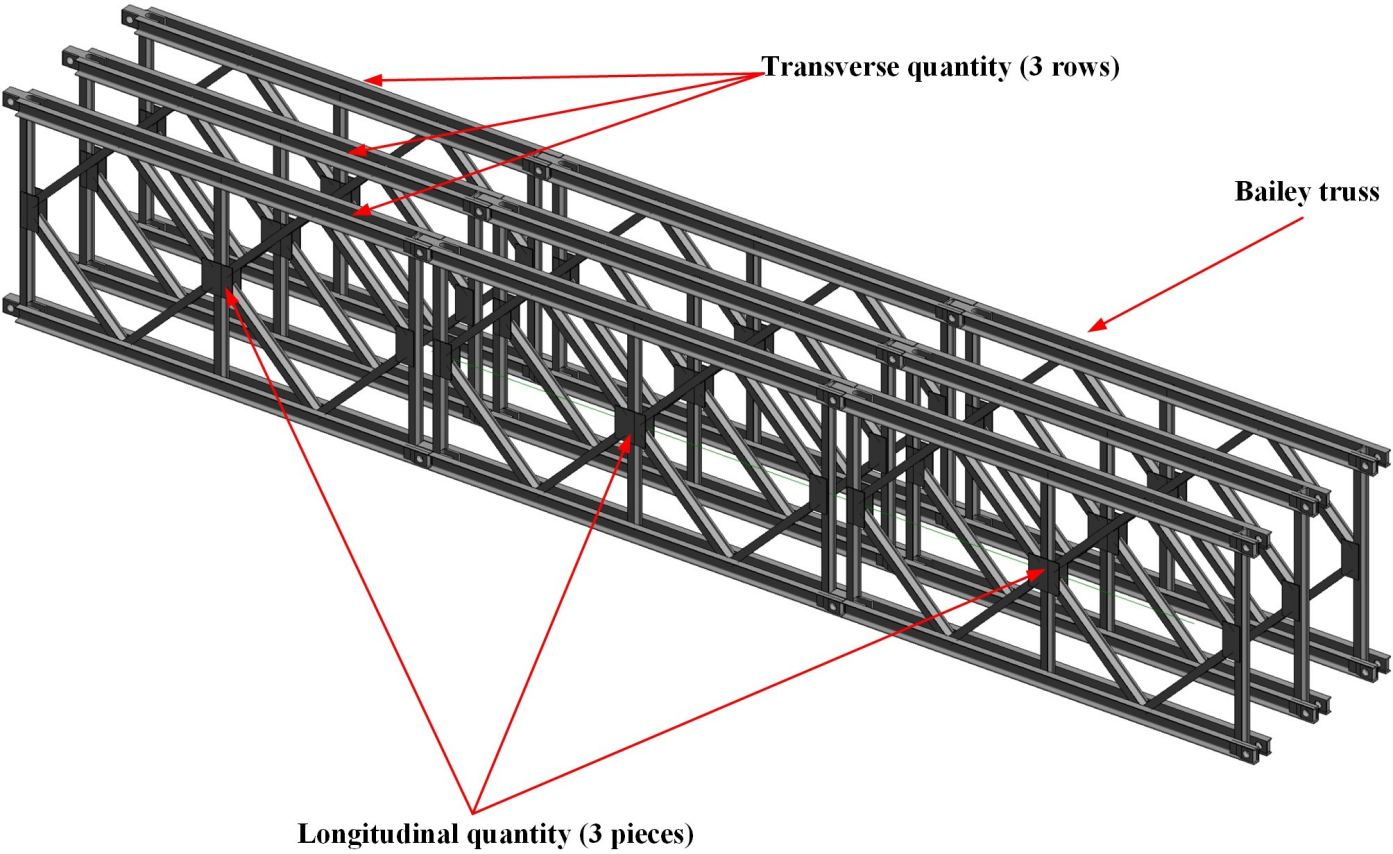
The parametric model of the Bailey truss.

The Bailey truss is a crucial component used for setting up support platforms and other support structures. Though the structural dimensions of the Bailey truss are fixed, the larger structures or platforms can be formed by assembling multiple units. Therefore, it is appropriate to use the longitudinal and transversal quantities of the Bailey truss as control parameters for parametric modeling. Thus, by configuring a single Bailey truss model as a parametric family, the parametric model of the Bailey truss can be established by modifying the longitudinal and transversal quantities. The aforementioned parametric model is shown in Fig 9.

### 2.6 Continuous beam bridge and overall construction site models

#### 2.6.1 Continuous beam bridge model

**Fig 10:**
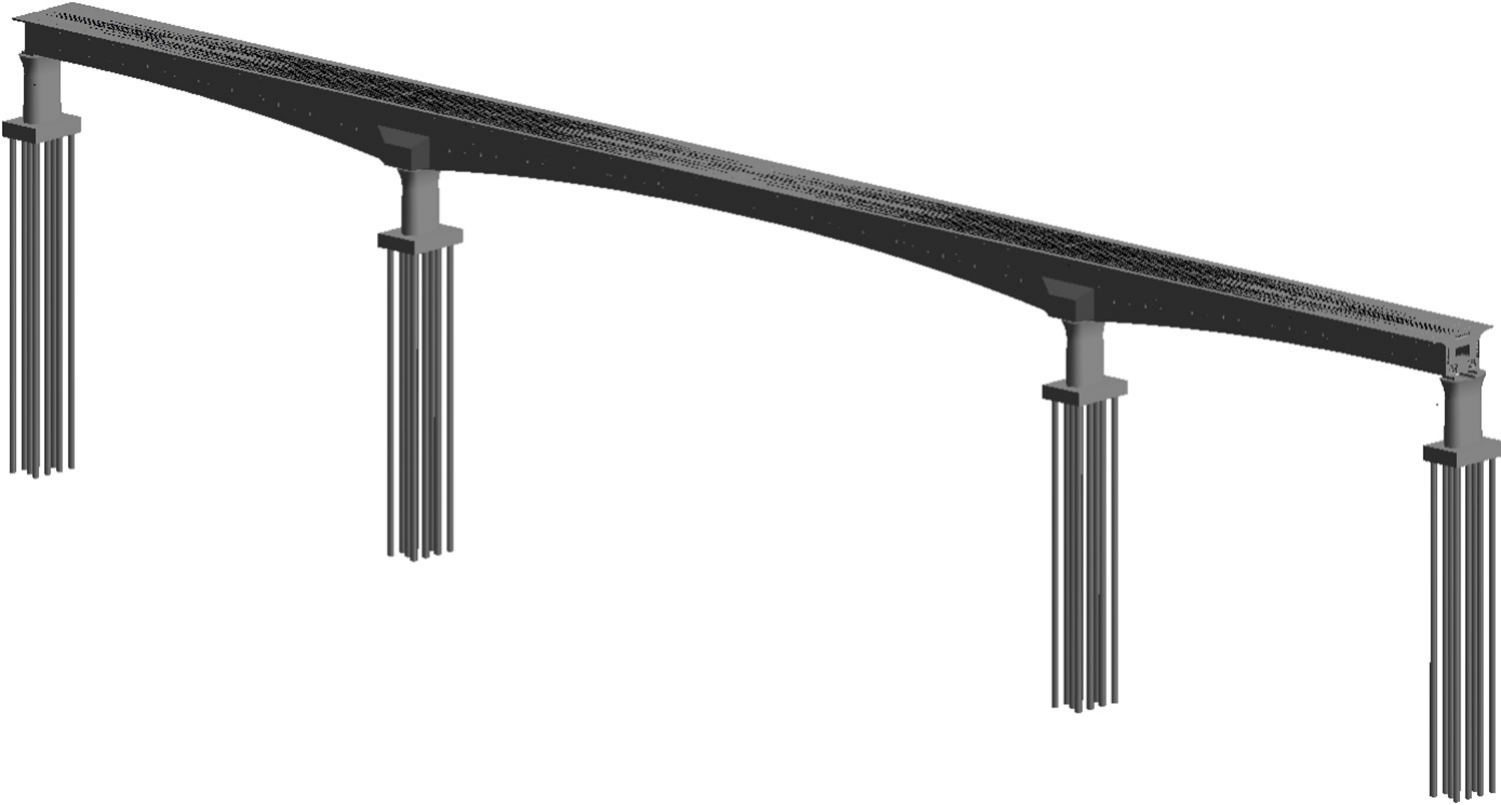
The overall structural model of HSR continuous beam bridge.

Based on the construction drawings and scheme, the overall structural model of the HSR continuous beam bridge is established on the foundation of the bridge structure’s parametric family, which is shown in Fig 10. For the upper structure of the continuous beam, all segments except for the 0th block are modeled based on the parametric family. Once the construction segment information is imported, the upper structure model is directly generated. The substructure is parametrically modeled based on the integrated family of piers, abutments, and pile foundations.

In HSR bridge construction, continuous beam bridges at different locations along the same alignment usually adopt identical structural forms and construction methods. Therefore, the aforementioned parametric modeling method for (73+128+73) m continuous beam bridge can be applied to other bridges of the same type. This approach can shorten the modeling time for similar models, reduce repetitive work, and ensure consistency in geometric and material information, thereby realizing the true potential of parametric modeling.

#### 2.6.2 Overall construction site model

**Fig 11:**
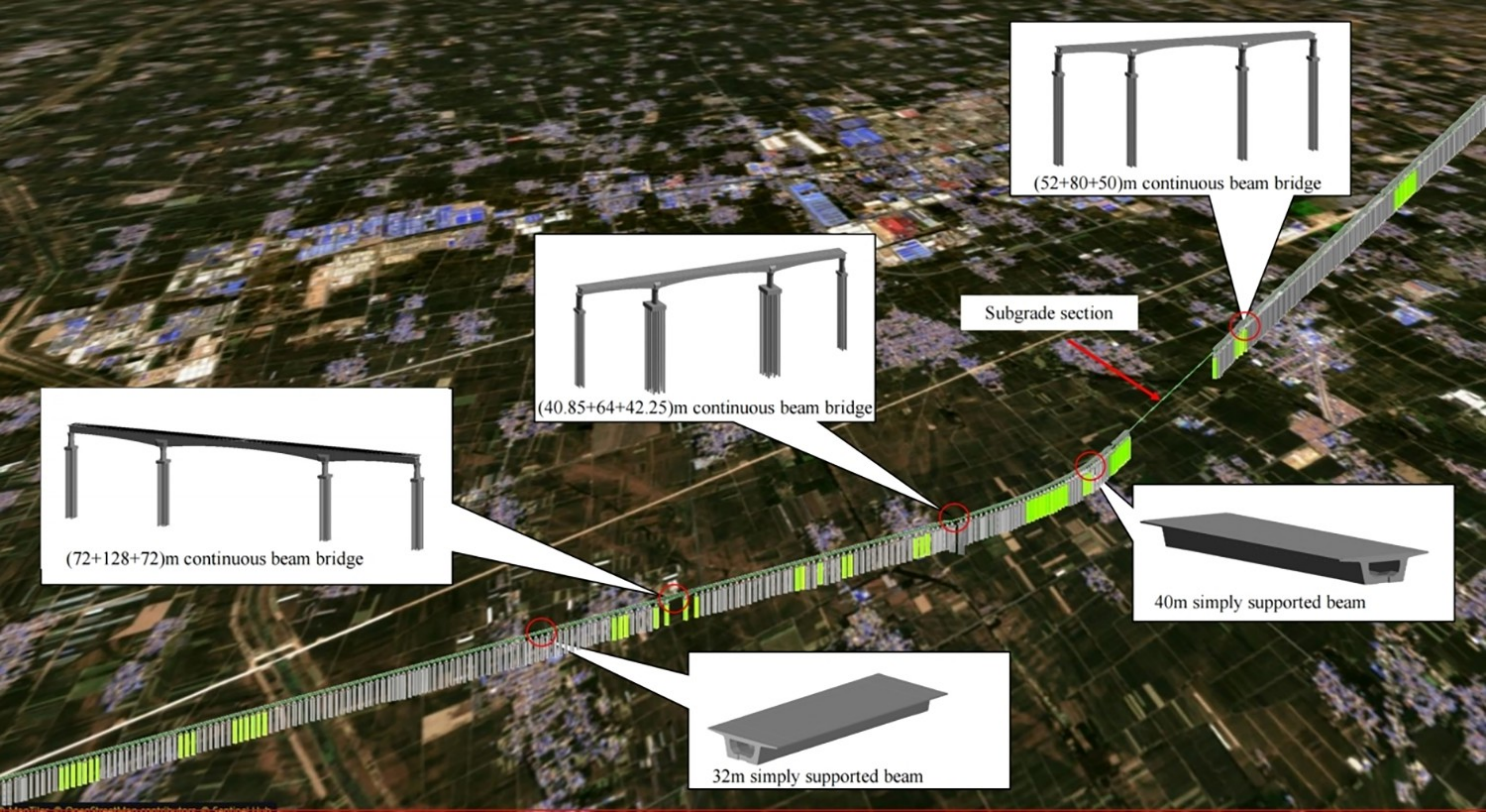
The overall construction site model (note that the figure uses map data from Sentinel Hub and their data sources; see collections.sentinel-hub.com/sentinel-s2-l2a-mosaic-120/).

On the basis of the completed continuous beam bridge model, the overall construction site model can be established by adding the structure model (including simply supported bridge, subgrade, etc.) and construction site model (including farmland, highway, etc.), as shown in Fig 11. According to the overall construction site model, the large-span continuous beam bridge structure can be visually displayed. The visual expression effect of the bridge construction scheme can be improved, and the efficiency and accuracy of construction disclosure can be further enhanced as well.

## 3. Engineering application during the construction phase

Prestressed duct construction and rebar clash detection are crucial aspects in the construction of large-span concrete continuous beam bridges. To enhance the precision of prestressed duct layout and eliminate the clash point between the rebar and prestressed steel strand, engineering application research has been conducted on the use of BIM models.

### 3.1 Prestressed duct layout

In the process of prestressed concrete construction, the prestressed duct layout should be carried out according to the designed coordinates before the steel strands are set up. This ensures the correct profile of the prestressed steel strands, allowing the prestressed concrete box girder to be fully compressed once constructed. Thus, the safety of site construction and the service life of the continuous beam bridge are guaranteed.

Currently, the prestressed duct layout primarily relies on 2D construction drawings, leading to two main issues in construction: 1) Bridge construction drawings typically include prestressed duct layouts for only a portion of the construction sections, making it time-consuming and laborious to guide the construction;2) When the prestressed duct is arranged as a spatial curve, measurement based on the planar and vertical curves in the drawings is required, which involves a significant amount of work and is prone to errors.

**Fig 12:**
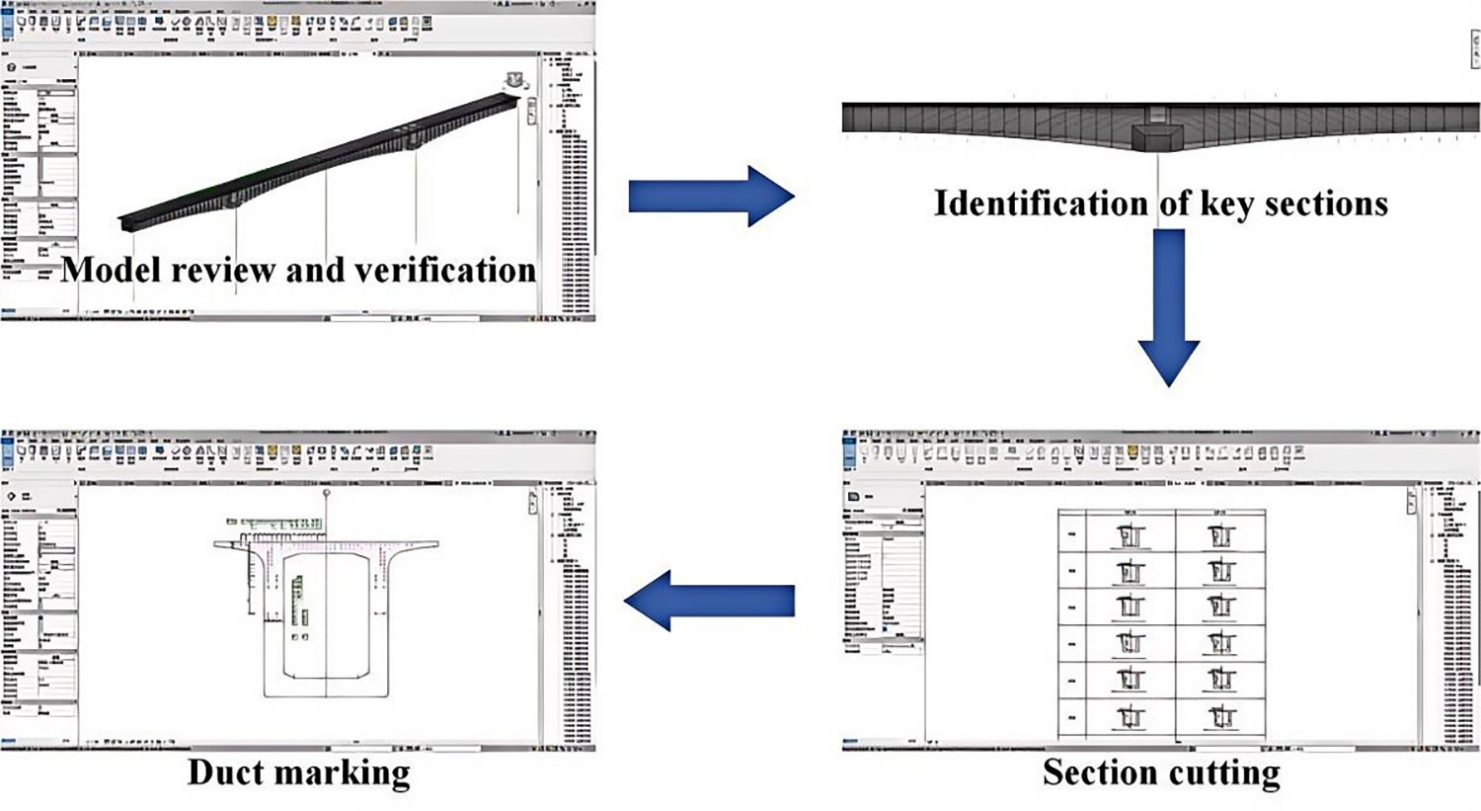
The parametric model of the Bailey truss.

To address the issues in the prestressed duct layout process, the BIM models are utilized to optimize the layout construction, and the process is demonstrated in Fig 12. It includes the following three steps: 1) Review and verify the bridge superstructure model and the prestressed duct model; 2) Identify key sections, including the end sections of the box girder segments and the start/end bend sections of the prestressed steel strands; 3) Cut the key cross-sections; 4) Mark the positions of ducts, subsequently generating the prestressed duct layout diagrams.

Engineering practice shows that the precision of space curve layout can be improved by producing layout diagrams for all key cross-sections. This measure can further compensate for the issues, which stem from the reliance on the personal experience of construction personnel, leading to low work accuracy and efficiency.

### 3.2 Rebar clash detection

Traditional rebar design employs 2D methods, which lack the capability for 3D visualization. Consequently, the resolution of rebar clash within components largely relies on the spatial imagination of the designers. It is well-known that bridge components contain an extensive number of rebars. Manual clash detection is not only time-consuming and laborious, but also the effect is not ideal. Suppose the rebar clash is transferred from the design stage to the construction stage. In that case, it will inevitably cause rework and construction delay, which is the existing pain point in the traditional rebar design of bridge.

By employing BIM technology and adhering to the principles of rebar arrangement, 3D modeling of rebar and steel strands can be accomplished. The clash detection before the rebar construction can effectively solve problems such as recutting and arrangement of rebar in the construction process, so as to avoid the delay of the construction period. The clash detection algorithm developed in this study is interpreted below:

1. 3D modeling of rebar and steel strands
  - Detailed 3D models of rebar and steel strands are created using BIM software.
  - Principles of rebar arrangement are followed to ensure accurate representation.
2. Clash detection process
  - The 3D models are imported into the clash detection module of the BIM software.
  - Tolerance levels for acceptable spatial clearances are defined.
  - The software algorithmically checks for intersections and conflicts between rebar and prestressed steel strands.
3. Automated detection and reporting
  - The software generates a clash report identifying all detected conflicts.
  - Each clash is categorized based on severity and location within the bridge model.
4. Resolution and iteration
  - Conflicts are resolved by adjusting the model parameters.
  - The clash detection process is iteratively repeated to ensure all issues are addressed before construction.

**Fig 13:**
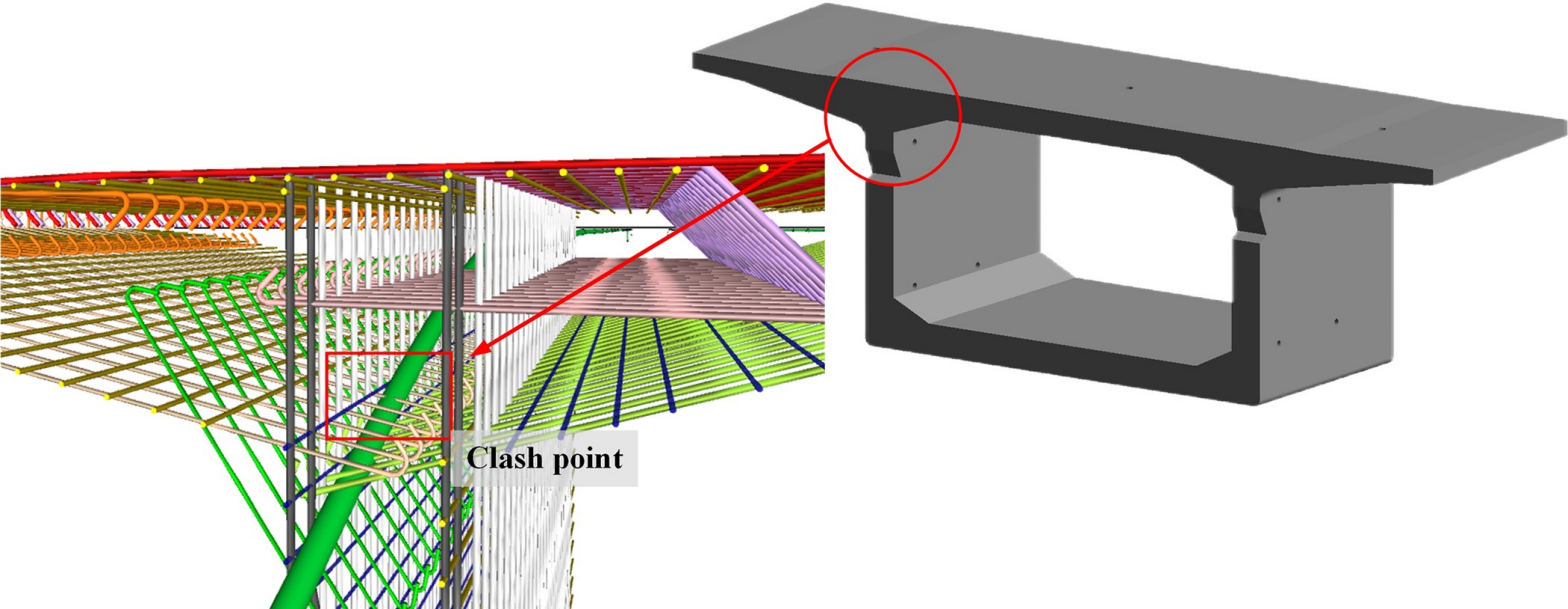
The clash result.

A block is selected for clash detection in this study. According to the aforementioned algorithm, the clash result between the rebar and the prestressed steel strands is illustrated in Fig 13.

## 4. Quantitative analysis

The effectiveness of the proposed parametric modeling method is presented in this section by conducting the quantitative analysis. The Caoxian-Shangqiu Grand Bridge is the HSR continuous beam bridge, which is suitable with the parametric modeling method, and the characteristics of a large span and complicated structure can reflect the advantages of the proposed method, as well. The quantitative analysis can be conducted in modeling efficiency, construction timeline, and accuracy improvement in the early stage of construction. Though the construction costs cannot be analyzed accurately before completion, the saved costs can still be supposed through the reduction of the modeling and construction time and the raising of construction accuracy by applying the proposed method. Therefore, the modeling efficiency and construction timeline are compared in this section. And the quantitative analysis of construction costs will be carried on systematically after the construction of the Caoxian-Shangqiu Grand Bridge in our next paper.

### 4.1 Modeling efficiency

To demonstrate the efficiency gains from our proposed parametric modeling method, we conducted a comparative analysis between traditional modeling techniques and our approach. The results indicated a 40% reduction in modeling time, enhancing the speed of project execution. Table 1 below illustrates the time spent on modeling various components using both methods.

**Table 1:** The comparison of modeling time.

Component	Traditional Method (hours)	Parametric Method (hours)	Reduction (%)
Total Modeling Time	100	60	40

### 4.2 Construction timeline

The application of our parametric modeling approach facilitated a 10.1% reduction in the construction timeline. This acceleration is particularly significant in high-speed railway projects where timelines are critical. The timeline data showing the project duration comparison is summarized in Table 2.

**Table 2:** The comparison of construction timeline.

Project Phase	Traditional Duration (weeks)	Parametric Method Duration (weeks)	Reduction (%)
Total Project Duration	69	62	10.1

### 4.3 Accuracy improvement

The accuracy of the project was significantly enhanced through the use of the parametric modeling method. The precision in the component placements, prestressed duct layout, and rebar arrangements of the HSR continuous beam bridge improved by 10%, resulting in higher quality construction and reduced need for adjustments on-site. The detailed and accurate models also facilitated better communication and understanding among stakeholders, contributing to smoother project execution.

In the research condition of this study, the modeling time and total project duration have been reduced by 40% and 10.1%, respectively. And the accuracy improvement has been increased by 10%, as well. According to the aforementioned improvements obtained, it can be inferred that the construction costs are also saved by applying the proposed method. As a consequence, the effectiveness of the parametric modeling method can be proved.

## 5. Conclusions

In this study, we developed a parametric rapid modeling method of HSR continuous beam bridge based on BIM. The Caoxian-Shangqiu bridge of Xiong’an-Shangqiu HSR is taken as the engineering application. The main conclusions are as follows:

1. A parametric modeling method for HSR continuous beam bridges has been proposed based on BIM, which represents a significant departure from conventional design methods. By introducing the BIM parametric family of continuous beam components and key construction machinery, a reduction in modeling workload and an enhancement in modeling efficiency can be achieved. This approach allows for rapid adaptations to design changes and complex conditions, offering a more dynamic and flexible solution for bridge engineering.
2. The application of BIM models in the layout of prestressed ducts has been explored. By exporting the layout drawings of ducts for all key cross-sections, an improvement in the precision of spatial curve prestressed duct layouts is attainable. The difficulty of complex prestressed duct layouts can be solved by the developed method, and the layout precision and efficiency are improved as well.
3. The rebar clash detection is carried out based on the parametric modeling method. And the detection before the rebar construction can effectively solve the problems such as recutting and arrangement of rebar in the construction process, so as to avoid delay in the construction period and save costs in practice.
4. The quantitative analysis is conducted to evaluate the effectiveness of the parametric modeling method proposed in this paper. The modeling efficiency and construction timeline are compared between the traditional method and the proposed one. In the research condition of this study, the modeling time and total project duration have been reduced by 40% and 10.1%, respectively. And the accuracy improvement has been increased by 10%, as well. According to the aforementioned improvements obtained, it can be inferred that the construction costs are also saved by applying the proposed method. As a consequence, the effectiveness of the parametric modeling method can be proved for practical applications.

To sum up, the parametric modeling method developed and applied in this study not only showcases a significant departure from conventional design methods but also offers practical, scalable solutions that enhance the efficiency and safety of high-speed continuous beam bridge projects, thereby further raising the informatization and intelligence level of HSR bridge construction. The parametric modeling method proposed in this study can be applied to many other similar construction projects and provide relevant references in practice and theory.
